# The neurobiological effects of mind–body exercise: a systematic review and meta-analysis of neuroimaging studies

**DOI:** 10.1038/s41598-023-37309-4

**Published:** 2023-07-06

**Authors:** Yvonne M. Y. Han, Melody M. Y. Chan, Coco X. T. Choi, Maxwell C. H. Law, Daniel Kwasi Ahorsu, Hector W. H. Tsang

**Affiliations:** 1 Department of Rehabilitation Sciences, The Hong Kong Polytechnic University, Hung Hom, Kowloon, Hong Kong SAR, China; 2 Queensland Brain Institute, The University of Queensland, St Lucia, QLD 4072 Australia; 3 Department of Special Education and Counselling, The Education University of Hong Kong, Hong Kong SAR, China

**Keywords:** Neuroscience, Psychology, Health care

## Abstract

The neurobiological effects of mind–body exercise on brain activation, functional neural connections and structural changes in the brain remain elusive. This systematic review and coordinate-based meta-analysis investigated the changes in resting-state and task-based brain activation, as well as structural brain changes before and after mind–body exercise compared to waitlist or active controls based on published structural or functional magnetic resonance imaging randomized controlled trials or cross-sectional studies. Electronic database search and manual search in relevant publications yielded 34 empirical studies with low-to-moderate risk of bias (assessed by Cochrane risk-of-bias tool for randomized trials or Joanna Briggs Institute’s critical appraisal checklist for analytical cross-sectional studies) that fulfilled the inclusion criteria, with 26 studies included in the narrative synthesis and 8 studies included in the meta-analysis. Coordinate-based meta-analysis showed that, while mind–body exercise enhanced the activation of the left anterior cingulate cortex within the default mode network (DMN), it induced more deactivation in the left supramarginal gyrus within the ventral attention network (uncorrected ps < 0.05). Meta-regression with duration of mind–body practice as a factor showed that, the activation of right inferior parietal gyrus within the DMN showed a positive association with increasing years of practice (voxel-corrected p < 0.005). Although mind–body exercise is shown to selectively modulate brain functional networks supporting attentional control and self-awareness, the overall certainty of evidence is limited by small number of studies. Further investigations are needed to understand the effects of both short-term and long-term mind–body exercise on structural changes in the brain.

PROSPERO registration number: CRD42021248984.

## Introduction

Mind and body practices are complementary health approaches that focus on facilitating brain-behavior connections, which aim to promote health and well-being by enhancing the ability of the mind to regulate our physical bodies for optimal daily functioning^1,2^. Among many mind and body practices, mind–body exercise (i.e., Tai Chi, qigong and yoga) is a specific type of practice that incorporates meditation into the execution of movement routines to improve body balance and the flexibility and strength of the musculoskeletal structures^3^. Unlike conventional physical exercise that specifically emphasizes the awareness of bodily movements^4^, mind–body exercise emphasizes the coordination between breathing, bodily sensation awareness, and bodily movement execution^5,6^. According to a National Health Interview Survey conducted in the United States^7^, mind–body exercise is listed as one of the most common complementary health approaches among adults. Due to its popularity, researchers have continuously studied the beneficial effects of mind–body exercise on human health. Numerous meta-analytic reviews show that mind–body practices are effective in promoting motor, cognitive and affective functioning of both healthy and clinical populations. For instance, Tai Chi has been shown to be effective at improving motor control in patients with stroke^8^ and Parkinson’s disease^9^, promoting psychological well-being in older adults^10^ and enhancing cognitive functions in both healthy^11,12^ and clinical^13,14^ elderly populations. Similar positive clinical effects have been observed when qigong^15–18^ or yoga^19–21^ is performed.

Given the promising clinical effects of mind–body exercise, the neurobiological mechanisms associated with the observed behavioral changes have been rigorously studied in recent years^22–24^. Specifically, functional magnetic resonance imaging (fMRI) has been widely adopted to investigate how mind–body exercise changes brain activation patterns, connections between different regions^25^ and brain structures, and some studies have shown that mind–body exercise induces brain changes in the frontoparietal regions. For example, Eyre, Acevedo^26^ showed that a 12-week yoga training program significantly reduced depressive symptoms and improved visuospatial memory in older adults with mild cognitive impairment compared to conventional cognitive training, with enhanced visuospatial memory performance significantly correlated with a reduction in the functional connectivity between the superior and medial parietal cortices that implies greater neural efficiency^26^ for visual attention and working memory^27^. Greater activation in the left ventrolateral prefrontal cortex (PFC), a brain region known for inhibitory control^28^, was found in long-term yoga practitioners compared to age-matched healthy individuals without regular yoga practice when these individuals were presented negative emotional stimuli^29^. Tao, Chen^30^ showed that 12-week Tai Chi and qigong practices in older adults increased regional spontaneous neuronal activity in the dorsolateral PFC and the medial PFC, respectively, which was associated with enhanced memory performance. Moreover, significant increases in both whole-brain white matter^31^ and hippocampal gray matter^32^ are evident after long-term mind–body practice. However, statistically negative results have also been reported; some studies reported no statistically significant exercise-induced changes in brain activation or functional connectivity patterns were found between the yoga^33^/Tai Chi^34^ practice group and the control group.

Notably, the experimental protocols and fMRI outcome measures vary substantially across the existing empirical studies, which might contribute to inconsistent results. For instance, the duration of mind–body practice in different study protocols is highly heterogeneous. Some studies have investigated the neurobiological effects of long-term mind–body practice^33,35^, but other researchers have examined how several weeks of mind–body practice potentially modify neural activities^26,34,36^. Moreover, the nature of the control group also varies. In addition, these empirical studies adopted different methods to analyze fMRI data. Some studies investigated the changes in spontaneous neuronal fluctuations before and after mind–body exercises^37–39^, whereas others investigated the changes in neural connectivity in different regions of interest, including the default mode network (DMN)^40^ and parietal regions^26^. Collectively, these differences might complicate interpretations of the overall results, hence limiting our understanding of the neurobiological effects of mind–body practice. Systematic reviews of previously published studies and neuroimaging meta-analyses might help us address the limitations described above. Specifically, using seed-based d mapping with permutation of subject images (SDM-PSI)^41^, the between-study experimental design heterogeneity discussed above could be controlled by covariate analyses and further explored with meta-regression. In addition, registering consistently coactivated brain regions identified through the meta-analysis on a standardized brain atlas may be possible^42^, which might help us identify which functional brain networks are consistently modulated by mind–body practice. Finally, the overall study power for the identification of consistently coactivated brain regions by mind–body practice may be enhanced by performing a meta-analysis. Given the aforementioned research gaps, this study aimed to investigate the neurobiological changes (i.e., brain activation, neural connections and structural changes in the brain) associated with mind–body exercise by qualitatively and quantitatively examining currently available fMRI data.

## Methods

### Literature search

The Preferred Reporting Items for Systematic Reviews and Meta-analysis (PRISMA) guidelines^43^ were used to guide the implementation of this review (PROSPERO registration number: CRD42021248984; see Table S1 for the PRISMA checklist and access the review protocol via https://www.crd.york.ac.uk/prospero/display_record.php?RecordID=248984). All authors first decided on the keywords and electronic databases to search, followed by the first title/abstract/keyword search of the electronic databases Scopus, Embase, ScienceDirect and PubMed conducted on April 2022 using the keywords (“mindful*” OR “mind–body” OR “yoga” OR “tai chi” OR “qigong”) AND (“magnetic resonance imaging” OR “functional magnetic resonance imaging” OR “MRI” OR “fMRI”) to identify relevant papers. Full search strategies were reported in Table S2. An updated search using the same keywords and search engines was performed in June 2023 to ensure the most up-to-date articles were included in the analysis. References from a previously published review^44^ were also screened. No limitations on the dates of publication were set.

### Study inclusion for the systematic review and meta-analysis

The systematic review included published randomized controlled trials (RCTs) and cross-sectional studies investigating the effects of mind–body exercise compared to regular physical exercise (active control)/waitlist control groups. After removing duplicated records, titles and abstracts of the records were screened. Records that were (1) not empirical studies (e.g., review articles, book chapters, conference proceedings and editorials), (2) nonhuman studies and (3) not involving mind–body practice as an intervention were excluded. During the full-text screening, studies that (1) investigated the brain activations during meditative state only, (2) did not report standard stereotactic coordinate space results (either Talairach or MNI), and (3) did not compare the effects of mind–body exercise with a physical exercise/waitlist control group were excluded. Only studies that fulfilled all inclusion criteria were included in the systematic review. For meta-analysis, only fMRI studies comparing whole-brain seed-based resting-state functional connectivity (rsFC)^45^ between mind–body exercise and active/waitlist control groups were included. The whole screening process was conducted independently by the co-first, second and third authors and recorded separately on each Excel spreadsheet. The first author decided to include or exclude papers when discrepancies appeared. Corresponding authors of the included papers were contacted if there was missing or unclear information.

### Data extraction and recoding

Data were independently extracted from the included papers and recorded by the second and third authors. The extracted data were recorded in an Excel spreadsheet independently by the co-first author, second and third authors and were cross-checked by the first author. Demographic characteristics (i.e., participants’ diagnoses, mean age, ratio of sex), experimental details (i.e., types of mind–body exercise, average duration of practice in years, and control group), and outcome measures (i.e., the MRI paradigm and analysis methods, main outcomes in the active group) were extracted. In particular for studies included in the meta-analysis, as we aimed to pool the peaks with significant group differences between mind–body exercise group and control groups reported in previous studies^46,47^, seed center coordinates and peak coordinates of between-group differences were extracted from each study.

Data recoding was performed to facilitate covariate analyses in the coordinate-base meta-analysis. First, to recode the seed center coordinates of individual studies in a standardized manner such that covariate analyses were possible, the seed center coordinates were registered within the seven resting-state brain networks reported by studies that used large-scale data from human cerebral^48,49^, cerebellar^50^, and striatal^51^ parcellation studies. The seven functional networks included the default mode network (DMN; coordinates other task-positive networks), somatomotor network (SMN; for motor control and execution), frontoparietal network (FPN; coordinates goal-directed behavior), dorsal attention network (DAN; for top-down attention control), ventral attention network (VAN; detects salient stimuli), limbic network (LIM; for emotional processing), and visual network (VIS; processes incoming visual information). Second, regarding the control group conditions, given some studies utilized control groups with participants performing regular physical exercise (e.g. treadmill walking/usual physical exercise regime) while other studies adopted waitlist control groups (e.g. not doing mind–body exercise while also not engaging in any kinds of physical exercise regime), studies with physical exercise as a control condition were recoded as having an “active control group”, while studies with waitlist control groups were recoded as having a "waitlist control group”.

### Quality of reporting appraisal and risk of bias within the studies

The revised Cochrane risk-of-bias tool for randomized trials (RoB 2)^52^ and Joanna Briggs Institute’s (JBI) critical appraisal checklist for analytical cross-sectional studies^53^ were used to evaluate the methodological quality of the included RCTs and cross-sectional studies, respectively. Bias arising from the randomization process, bias due to deviations from intended interventions, bias due to missing outcome data, bias in measurement of the outcome, and bias in selection of the reported result were assessed using RoB 2. Three risk of bias categories (low, high or some concerns) were possible for each domain. The overall rating of the five domains yielded the “overall risk-of-bias judgment” for each of the included studies. Using the JBI critical appraisal checklist, the included cross-sectional studies were qualified, as suggested by Ma, Wang^54^. By counting the number of “yes” responses from the critical appraisal items among the total items of the study, the quality of each study was then assessed. Criteria with “not applicable” responses were excluded, while “unclear” responses were coded as “no” and did not fulfill the quality criteria. “Yes” scores for 0–33% of the JBI items were assigned a “low” overall quality rating, while 34–66% “yes” scores from the JBI questions were assigned a “medium” rating, and 67% or more “yes” scores for the JBI items were assigned a “high” rating.

### Data analysis

Narrative synthesis was performed for studies included in the systematic review. Effect sizes (Cohen’s d) and 95% confidence intervals (CI) were calculated using the formula reported in Lipsey and Wilson^55^ for all included experiments based on the significance threshold adopted by individual papers. the SDM-PSI software version 6.21^41^was used to pool the brain imaging data for the meta-analysis. The recommended data preprocessing pipeline was used^41^. Briefly, preprocessing of extracted fMRI coordinate data was performed with anisotropy = 1, isotropic full width at half maximum (FWHM) set to 20 mm, and a voxel size of 2 mm on a gray matter mask. The between-group analysis was performed with rsFC seed network as a covariate to investigate the effects of mind–body exercise on rsFC. Meta-regression analyses with mean age of participants (years) mean years of mind–body practice as independent variable and peak coordinates of between-group differences (mind–body exercise *vs.* control group) as dependent variable was performed to explore how duration of mind–body practice modulates the changes in brain activation. Regarding the significance threshold, we first identified significant clusters with the threshold p = 0.05 (uncorrected), Z > 1 and a cluster size (k) larger than 10 voxels, followed by performing voxel-wise error corrections using 1000 permutations to obtain the null distribution of cluster sizes that pass the threshold of p < 0.005, and that distribution was used to set a minimum cluster size^56^. Each of the local peaks within the significant clusters was classified based on their location within the seven functional networks to understand how mind–body exercise modulate the functional brain networks (i.e., visual, somatomotor, dorsal attention, ventral attention, frontoparietal, limbic and default mode networks). The heterogeneity between studies was indicated by I^2^ test with I^2^ = 25%, 50% and 75% corresponds to low, medium and high heterogeneity respectively^57^, which was calculated for statistically significant peak coordinates yielded from the between-group or meta-regression analyses. Harbord Egger bias tests^58^ were used to assess “small study effects”. Certainty assessment was conducted using the Grading of Recommendations, Assessment, Development and Evaluation (GRADE) approach^59^.

## Results

### Study selection

**Figure 1:**
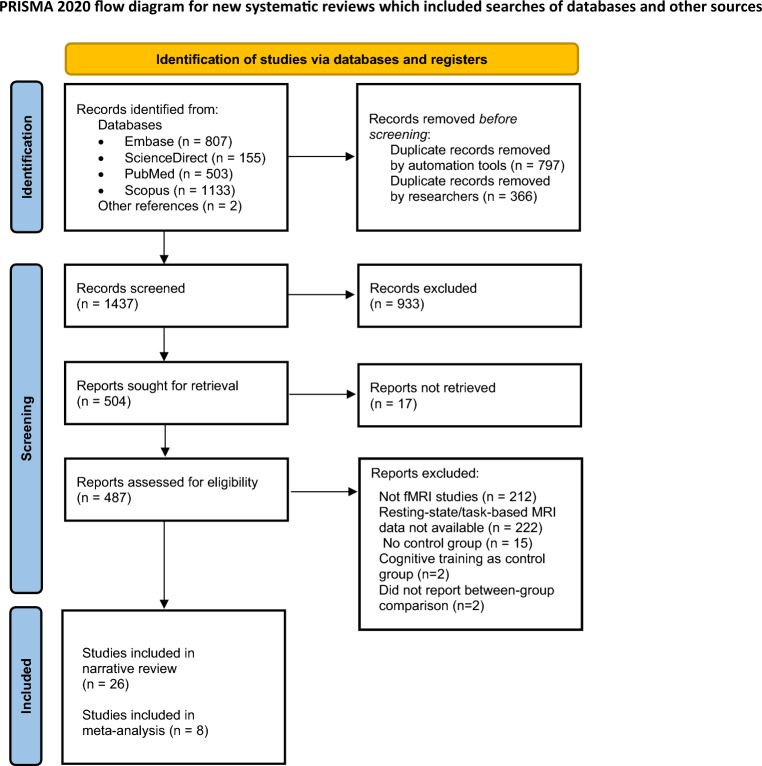
Flowchart of the article screening process.

A total of 2600 records were retrieved from multiple electronic databases and references. After the removal of duplicate records, 1437 abstracts were screened. After the exclusion of nonempirical non-fMRI studies and studies that did not involve mind–body interventions, the full text of 487 studies was screened for inclusion in the systematic review. Based on the inclusion and exclusion criteria, 34 fMRI studies (with 42 comparisons) were included, with 26 studies included in the narrative synthesis and 8 studies (with 13 comparisons) included in the meta-analysis. The flow of the article selection process is outlined in Fig. 1.

### Risk of bias within studies

**Figure 2:**
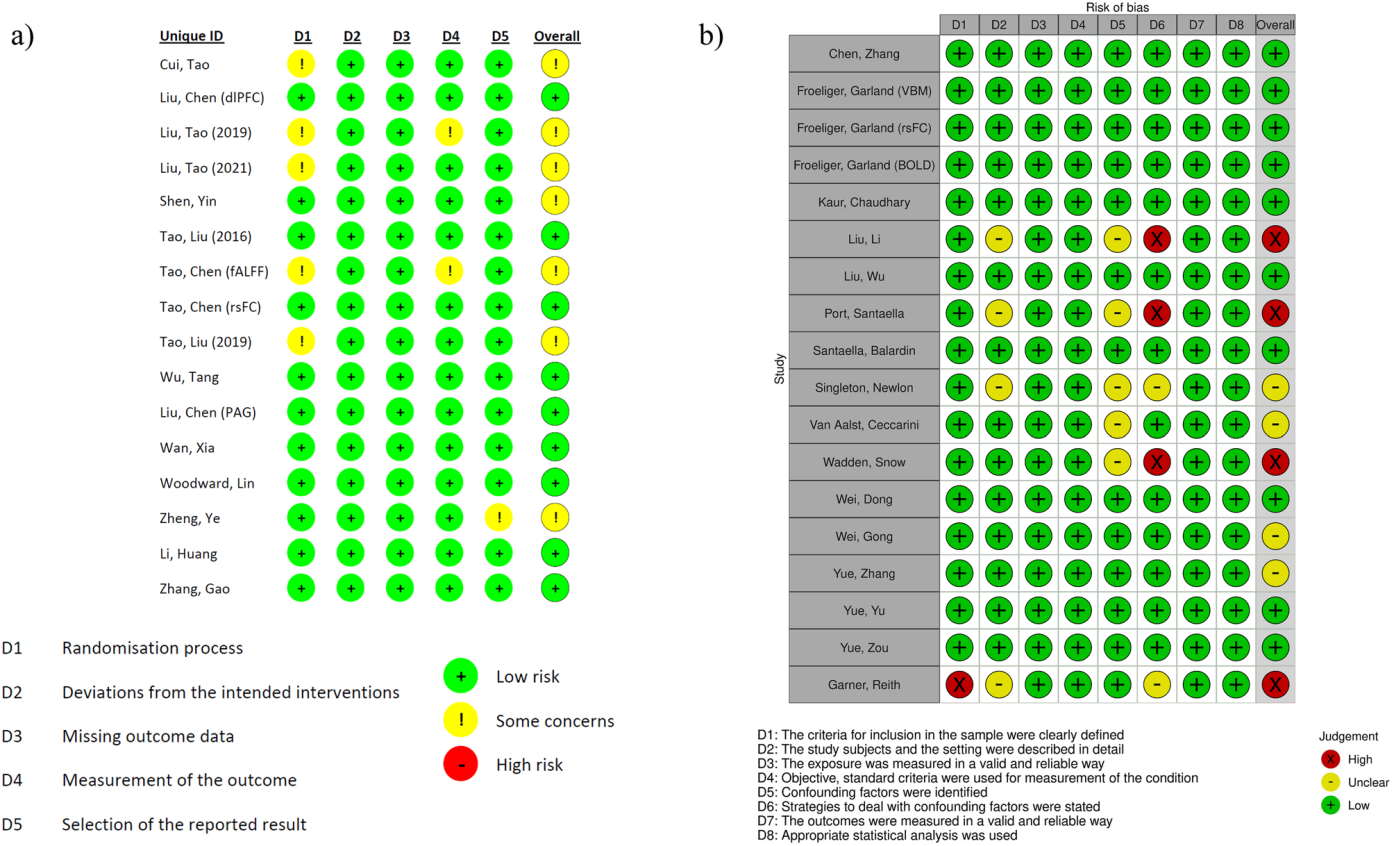
Risk of bias summaries: review authors’ judgements about each risk of bias item for each included (**a**) RCT and (**b**) cross-sectional study.

The summary of the authors’ judgment of the risk of bias of the included RCTs and cross-sectional studies is shown in Fig. 2. For the included RCTs (n = 16; Fig. 2a), nine studies had a low overall risk of bias, while the remaining seven studies warranted some concerns. All studies showed a low risk of bias in deviations from the intended interventions and missing outcome data. One study showed unclear bias in selective reporting of results. Unclear bias in outcome measurement was noted in two studies, and an unclear randomization process was noted in five studies. For the included cross-sectional studies (n = 18; Fig. 2b), four studies were rated as having an unclear overall risk of bias, and four studies were rated as having a high overall risk of bias. All studies showed a low risk of bias in defining participants’ inclusion criteria (D1), the use of valid, reliable and objective measurement tools (D3 and D4), outcome measurement (D7) and the choice of statistical analysis (D8). Four of 18 studies showed an unclear risk of bias in the participants’ demographic characteristics (D2). Five of 18 studies showed unclear risk of bias in the identification of confounding factors (D5). Four studies did not report strategies to control for the stated confounding factors (D6).

### Study characteristics

Table 1 lists the demographic characteristics, experimental design, neuroimaging outcome measures and results of the 35 studies included in this review. Detailed narrative and quantitative syntheses are presented in the subsequent paragraphs.

**Table 1:** Effects of mind–body exercise on different neuroimaging outcomes (34 studies, 42 experiments).

Reference (year)	Participants’ details				Experimental protocol			Outcome measures				Statistical threshold^b^
	Population	N	Mean Age (years)	Sex (M:F)	Mind–body practice	Average duration of practice (years)	Control group	MRI paradigm	MRI analysis	Between-group results^a^ (rsFC seed; if applicable)	Effect size in *d* [95%CI]	
Experiments included in the narrative synthesis (26 studies, 29 comparisons)												
Chen^15^	Healthy	22	52.4	15:7	TCC	14	Passive	Resting	rsFC (ROI-ROI)	↓ R MFG–L MFG (DMN)	0.643 [0.00470, 1.28]	Family-wise-error corrected *p* < 0.05
		18	54.8	10:8								
Cui^61^	Healthy	12	21.8	2:10	TCC	0.167	Active	Resting	Graph theory network analysis	*Nodal clustering coefficient* ↑ L thalamus ↑ L olfactory cortex *Nodal efficiency* ↑ Bilateral posterior cingulate ↑ R cuneus *Nodal local efficiency* ↑ L thalamus ↑ R olfactory cortex	0.85 [0.01, 1.68]	Cluster-corrected, *p* < 0.05
		12	21.8	2:10								
Froeliger^73^	Healthy	7	36.4	6:1	Yoga	9.3	Regular physical activity	Resting	VBM	↑ GM volume in frontal, limbic, temporal, occipital, and cerebellar regions	1.16 [0.03, 2.30]	Cluster-corrected, *p* < 0.05
		7	35.5	6:1								
Froeliger^35^								Resting	Network-based rsFC	*DAN within-network* ↑ R anterior IPS–FEF ↑ R anterior IPS–L middle temporal cortex ↑ R posterior IPS–L middle temporal cortex ↑ R middle temporal cortex–L FEF		
Froeliger^29^								(1) Emotional picture viewing task (2) Stroop task with distractor of negative emotion	BOLD activation (ROI)	↓ R dlPFC (negative–neutral) during emotion viewing ↑ L vlPFC (negative–neutral) during Stroop task		
Garner^75^	Healthy	39	22.7	5:34	Yoga	0.2	Strengthening/stretching exercise	Resting	VBM	*VBM GM change* ↓ L insula ↓ R IFG ↓ L IPL, AG	− 0.86 [− 1.44, − 0.28]	*Cluster-corrected p* < 0.05
		32	22.9	1:31								
Kaur^71^	Healthy	13	27.2	6:7	Yoga	3	Active lifestyle	N-back task Stroop	BOLD activation and FC (ROI)	*Activation changes* *1-back* ↑ STG ↑ L inferior semi lunar lobule ↑ Precentral gyrus ↑ Insula ↑ Parahippocampal gyrus *2-back* ↑ MTG ↑ Left cerebellar tonsil *Color stroop* ↑ Inferior occipital gyrus ↑ Precuneus ↑ Parahippocampal gyrus ↑ Inferior semi lunar lobule ↑ Cerebellar tonsil ↑ STG *Task-based FC changes* *1-back* ↑ L lPFC–R cerebellum ↑ R lPFC–R Insula ↓ L IPS–R PCG/R Insula/L cerebellum *2-back* ↑ R FEF–L SFG ↑ L PFC–R cerebellum ↑ R IPS–Right R Cuneus cortex *Color stroop* ↑ FPN	0.81 [0.01, 61]	Family-wise-error corrected *p* < 0.05
		13	28.6	6:7								
Li^64^	Parkinson’s disease	32	62.7	17:15	TCC	1	Walking	Resting	Resting-state network connectivity	↑ Visual network associated with better functional balance performance	0.60 [0.0035, 1.20]	Voxel-wise corrected p < 0.05
		17	*n.r.*	*n.r.*								
Liu^100^	Osteoarthritis	28	n.r.	6:22	TCC	0.25	Cycling	Resting	Seed to whole-brain rsFC	*PAG as seed* ↑ L PAG–R MTG ↑ R PAG– R AG ↑ R PAG–L MTG ↓ L PAG–R MFG ↓ L PAG–L PCG ↓ R PAG–R mPFC ↓ R PAG–R NAc ↓ R PAG–bil. ACC	0.54 [0.0028, 1.08]	*Cluster-corrected p* < 0.05
		27		4:23								
		29	n.r.	5:24	BDJ	0.25	Cycling	Resting	Seed to whole-brain rsFC	*PAG as seed* ↑ R PAG–PAG–R AG ↓ R PAG–R mPFC	0.54 [0.0027, 1.07]	*Cluster-corrected p* < 0.05
		27		4:23								
Liu^72^	Healthy	27	65.1	10:17	TCC	10.0	Regular physical activity	Sequential decision task	BOLD activation and FC (ROI)	↑Fronto-striatal connectivity	0.55 [0.00290, 1.10]	Family-wise correction, *p* < 0.05
		26	64.2	10:16								
Liu^62^	MCI	20	66.2	5:15	BDJ	0.5	Walking	Resting	Seed to whole-brain rsFC	*LC as seed* ↑ L LC–R dlPFC ↑ R LC–R ACC ↓ L LC–Bilateral cerebellum exterior ↓ R LC–R inferior occipital cortex ↓ R LC–Bilateral precentral cortex ↓ R LC–Bilateral postcentral cortex ↓ R LC–L middle occipital cortex ↓ R LC–R cerebellum *VTA as seed* ↑ L VTA–Bilateral ACC ↑ R VTA–R TPJ	0.99 [0.304, 1.67]	Voxel-wise Uncorrected, *p* < 0.005
		17	64.3	7:10								
Port^97^	Healthy	8	66.4	3:5 3:5	TCC	17.4	Regular physical activity	(1) n-back task (2) Stroop task	BOLD activation (whole-brain)	*n-back* ↓ R frontal pole ↓ SFG *Stroop* ↓ R intralcalcarine cortex ↓ Lateral occipital cortex ↓ Occipital pole	1.07 [0.024, 2.12]	*Cluster-corrected p* < 0.05
		8	66.4									
Shen^37^	Healthy	12	21.8	2:10	TCC	0.16	Brisk walking	Resting	fALFF	↑ Lateral medial SFG ↑ R FFG ↓ R dorsal SFG ↓ R PCL	0.85 [0.0114, 1.68]	Family-wise-error corrected *p* < 0.05
		12	21.8	2:10								
Tao^63^	Healthy	21	62.4	8:13	TCC	0.25	Regular physical activity	Resting	Seed to whole-brain rsFC	↑ Bilateral hippocampus–Bilateral mPFC	0.597 [0.00370, 1.19]	*Cluster-corrected p* < 0.05
		25	59.8	6:19								
Tao^63^		16	62.2	6:10	BDJ	0.25				↑ Bilateral hippocampus–Bilateral mPFC	0.648 [0.00460, 1.29]	*Cluster-corrected p* < 0.05
		25	59.8	6:19								
Tao^30^	Healthy	21	62.4	8:13	TCC	0.25	Regular physical activity	Resting	fALFF	↑ dlPFC (slow-5 and low-frequency)	0.597 [0.00370, 1.19]	*Cluster-corrected p* < 0.05
		25	59.8	6:19								
Tao^30^		15	62.3	6:9	BDJ	0.25				↑ Bilateral mPFC (slow-5 and low-frequency)	0.661 95% C.I. [0.00490, 1.32]	*Cluster-corrected p* < 0.005
		25	59.8	6:19								
Tao^38^	MCI	20	66.2	5:15	BDJ	0.25	Walking	Resting	VBM, fALFF, seed to whole-brain rsFC	*VBM GM volume changes* ↑ R hippocampus *fALFF changes* ↑ mPFC (slow-4) ↑ dlPFC (slow-4) ↑ Bilateral ACC (slow-5) ↓ Bilateral lingual gyrus (slow-4) ↓ L hippocampus (slow-4) ↓ R STG *rsFC changes* ↑ R hippocampus–L mPFC ↑ R hippocampus–R angular gyrus	0.988 [0.304, 1.67]	*Cluster-corrected p* < 0.05
		17	64.3	7:10								
Wadden^70^	Healthy	19	35.9	3:16	Yoga	0.5	Regular physical activity	Emotional/non-emotional video viewing Happiness sadness anger	BOLD activation (whole-brain)	↑ L SPL ↑R anterior supramarginal gyrus ↑ Postcentral gyrus	1.12 [0.189, 1.67]	Voxel-wise uncorrected, *p* < 0.005
		12	32.6	6:6								
Wan^98^	Subclinical (cognitive decline)	26	67.4	9:17	BDJ	0.5	Regular physical activity	Resting	GM (ROI)	*GM change* ↑ R CA1 ↑ R presubiculum ↑ L parasubiculum	0.57 [0.00320, 1.135]	Cluster-corrected, *p* < 0.05
		24	64.7	12:12								
Wei^65^	Healthy	18	52.4	7:11	TCC	14.6	Sedentary lifestyle	Resting	ReHo	↓ L ACC ↓R SFC of dlPFC ↑ R postcentral gyrus	0.643 [0.00470, 1.28]	*Cluster-corrected* p < 0.05
		22	54.8	8:14								
Wei^39^	Healthy	18	52.4	7:11	TCC	14.6	Sedentary lifestyle	Resting	fALFF	↓ DMN ↓ Bilateral FPN ↓ Anterior cingulate-FPN network	0.643 [0.00470, 1.28]	*Cluster-corrected p* < 0.05
		22	54.8	8:14								
Woodward^76^	Early psychosis	21	21.5	0:21	Yoga	0.25	Aerobic exercise	Resting	GM (ROI)	Aerobic exercise > yoga: bilateral fusiform GM volume	0.65 [0.00490, 1.30]	*p* < 0.05
		18	22.3	0:18								
Wu^8^	Healthy	16	64.9	3:13	TCC	0.25	Regular physical activity	Task-switching paradigm	BOLD activation (whole-brain, ROI)	↑ L SFG ↑ R MFG	1.09 [0.337, 1.85]	Family-wise-error corrected *p* < 0.005
		15	64.9	0:15								
Yue^32^	Healthy	20	62.9	0:20	TCC	16.6	Walking	Resting	VBM, ReHo	*VBM* GM volume *changes* ↑ R ITG, L hippocampus and L cerebellum *ReHo changes:* ↑L hippocampus ↑ Fusiform gyrus	1.10 [0.448, 1.75]	voxel-wise uncorrected, *p* < 0.001
		22	62.4	0:22								
Yue^31^	Healthy	20	62.9	0:20	TCC	6	Walking	Resting	Graph theory network analysis	*White matter network* ↑ normalized clustering coefficient ↑ characteristic path length	0.624 [0.00430, 1.24]	(Correction not mentioned), *p* < 0.05
		22	62.4	0:22								
Zhang^99^	Subclinical (mood/anxiety symptoms)	9	24.2	2:7	TCC	0.17	Regular physical activity	Resting	fALFF	*fALFF changes* ↑ R MFG (orbital part) ↑ R MTG (temporal pole) ↑ R MOG	1.00 [0.0194, 1.98]	*Cluster-corrected p* < 0.05
		9	22.5	3:6								
Zheng ^74^	MCI	23	65.8	6:17	BDJ	0.5	Walking	Resting	VBM	*VBM* GM volume *changes* ↑ R PCG ↑ R MOG	0.78 [0.193, 1.394]	Voxel-wise corrected, *p* < 0.01
		23	64.9	11:12								
Experiments included in the meta-analysis (8 studies, 13 comparisons)												
Liu^34^	Healthy	28	58.6	n.r.	TCC	0.25	Waitlist control	Resting	Seed to whole-brain rsFC	Bilateral dlPFC (MNI coordinates: ± 36,27,29; FPN)	0.559 [0.00300, 1.11]	*Cluster-corrected p* < 0.05
		24	56.9									
Liu^34^		29	59.7		BDJ					Bilateral dlPFC (MNI coordinates: ± 36,27,29; FPN)	0.554 [0.00300, 1.11]	*Cluster-corrected p* < 0.05
		24	56.9									
Liu^34^		28	58.6		TCC		Walking			Bilateral dlPFC (MNI coordinates: ± 36,27,29; FPN)	0.559 [0.00300, 1.11]	*Cluster-corrected* p < 0.05
		27	61.3									
Liu^34^		29	59.7		BDJ					Bilateral dlPFC (MNI coordinates: ± 36,27,29; FPN)	0.554 [0.00300, 1.11]	*Cluster-corrected* p < 0.05
		27	61.3									
Liu^40^		21	62.4	8:13	TCC		Waitlist control			PCC (MNI coordinates: -2, -36, 37; DMN) mPFC (MNI coordinates: 1, 54, 21; DMN)	0.597 [0.00370, 1.19]	*Cluster-corrected p* < 0.05
		25	60.1	6:19								
Liu^40^		16	62.2	6:10	BDJ						0.65 [0.00460, 1.29]	*Cluster-corrected p* < 0.05
		25	60.1	6:19								
Liu^66^		16	65.19	8:18	TCC	10.4	Regular physical activity			dlPFC (MNI coordinates: 36,27,29; FPN)	0.95 [0.292, 1.61]	Voxel-wise uncorrected, *p* < 0.005
		25	63.92	9:16								
Tao^36^		21	62.38	8:13	TCC	0.25				Bilateral dlPFC (MNI coordinates: ± 36.27.29; FPN) Bilateral dlPFC (MNI coordinates: ± 36.27.29; FPN)	0.597 [0.00370, 1.19]	*Cluster-corrected p* < 0.05
		25	59.76	6:19								
Tao^36^		16	62.33	6:9	BDJ						0.648 [0.00460, 1.29]	*Cluster-corrected p* < 0.05
		25	59.76	6:19								
Santaella^67^		20	68.2	0:20	Yoga	15.1				PCC (MNI coordinates: 1, − 61, 38; DMN) mPFC (MNI coordinates: 1, 55, − 3; DMN)	0.640 [0.00470, 1.28]	Family-wise-error corrected *p* < 0.05
		20	66.5	0:20								
Singleton^68^		16	49.4	5:11	Yoga	18.3				PCC (MNI coordinates: − 9.6, − 51.3, 27.5; DMN)	0.956 [0.235, 1.68]	*Cluster-corrected p* < 0.01
		17	52.5	7:10								
van Aalst^33^		10	36.8	2:8	Yoga	4.8	Stationary cycling		WHOLE-brain rsFC	DMN (MNI coordinates: not specific) DMN (MNI coordinates: not specific)	1.75 [0.723, 2.79]	Voxel-wise uncorrected, *p* < 0.001
		10	34.6	2:8								
Yue^69^		20	62.9	0:20	TCC	6	Walking				0.624 [0.00430, 1.24]	Family-wise-error corrected *p* < 0.05
		22	63.3	0:22								

*N* number of participants, *TCC* Tai Chi Chung, *BDJ* Baduanjin, *VBM* voxel-based morphometry, *GM* grey matter, *VMHC* voxel-mirrored homotopic connectivity, *ReHo* regional homogeneity, *fALFF* fractional amplitude of low-frequency fluctuations, *BOLD* blood-oxygen-level-dependent, *rsFC* resting-state functional connectivity, *ROI* regions of interest, *DTI* diffusion tensor imaging, *DMN* default mode network, *DAN* dorsal attention network, *FPN* frontoparietal network, *SN* salience network, *MFG* middle frontal gyrus, *MFC* medial frontal cortex, *PFC* prefrontal cortex, *aPFC* anterior prefrontal cortex, *lPFC* lateral prefrontal cortex, *mPFC* medial prefrontal cortex, *dmPFC* dorsal medial prefrontal cortex, *dlPFC* dorsal lateral prefrontal cortex, *vlPFC* ventral lateral prefrontal cortex, *SFG* superior frontal gyrus, *ACC* anterior cingulate cortex, *PCC* posterior cingulate cortex, *IPS* intraparietal sulcus, *SPL* superior parietal lobule, *FEF* frontal eye field, *STG* superior temporal gyrus, *MTG* middle temporal gyrus, *ITG* inferior temporal gyrus, *LC* locus coeruleus, *VTA* ventral tegmental area, *FFG* fusiform gyrus, *PCL* paracentral lobule, *PCG* precentral gyrus, *AG* angular gyrus, *PAG* periaqueductal gray, *NAc* nucleus accumbens, *n.r.* not reported.

^a^Statistically significant results only; contrast of interest: mind-body practice group > control group, unless otherwise specified.

### Can mind–body exercise modulate resting-state brain activities?

Twenty-four studies showed that both long-term and short-term mind–body practice induced changes in resting-state brain activities (Table 1). Most of the studies reported that mind–body exercise is capable of modulating the synchronization between different brain regions. Among studies that reported changes in brain synchronization, 13 experiments (reported in 8 studies) were included in the meta-analysis based on the inclusion criteria. In addition, some studies have shown that mind–body exercise modulates spontaneous neuronal fluctuations.

Nine studies reporting changes in rsFC, as indexed by the statistical correlations of BOLD signals between selected brain regions, were included in the narrative synthesis only. These studies showed that mind–body exercise is capable of reducing rsFC of the cortical brain regions within the DMN^60^, visual network^61^, as well as between bilateral periaqueductal gray (PAG) and the right medial prefrontal cortex^63^, while increasing rsFC between the DMN and hippocampus^38,62^, between PAG and parietotemporal regions^63^, and increasing rsFC within the DAN^35^. The remaining three studies showed significant changes in local brain region synchronization, as indexed by changes in regional homogeneity^32,64^ and nodal efficiency^65^.

**Figure 3:**
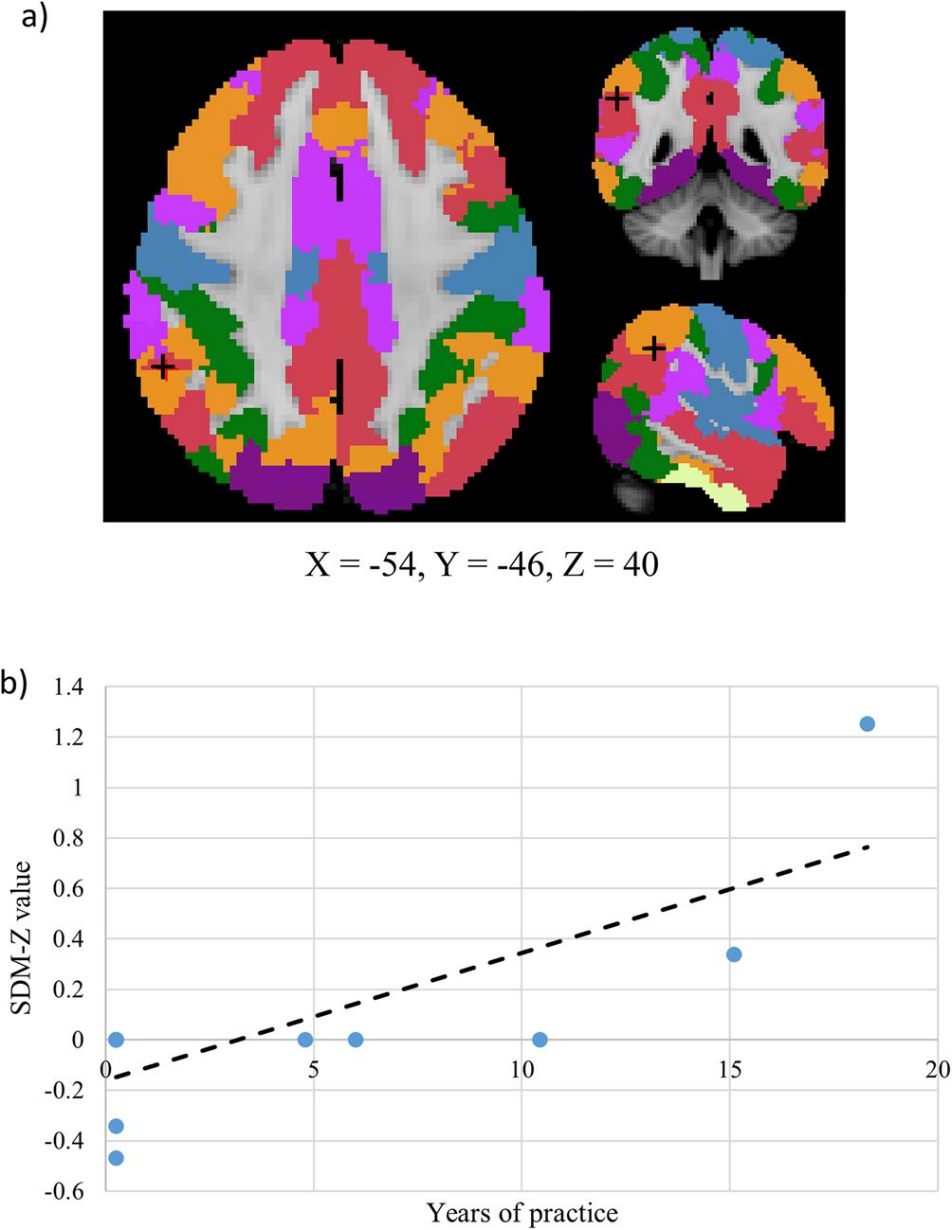
(**a**) The activation of right inferior parietal gyri (BA40, peak voxel indicated by ‘+’ symbol), which lies within the default mode network (red), increases with years of mind–body practice (voxel-corrected p < 0.005). (**b**) A scattered plot showing the positive relationship between the activation of right inferior parietal gyri and years of mind–body practice.

Thirteen experiments reported in eight studies were included in the SDM-PSI coordinate-based meta-analysis^33,34,36,40,66–69^. With seed location and control condition as covariates, increased activation was observed in the DMN with a peak at the left anterior cingulate gyri and decreased activation was observed in the VAN with a peak at the left supramarginal gyrus in the mind–body exercise group when compared to the control group (uncorrected p < 0.05; Table 2). These peaks did not survive voxel-corrected significance tests. Meta-regression between brain activation and years of mind–body practice showed that, the activation difference between treatment and control group of right inferior parietal gyri (BA40) increases with increasing years of mind–body practice (voxel-corrected p = 0.003; Fig. 3). Meta-regression of mean age of participants did not reveal significant correlation with brain activation patterns at the whole brain level (p > 0.05). Regarding heterogeneity and small study effects, the left anterior cingulate gyri (I^2^ < 0.01%; Harbord Egger bias tests p-value = 0.93), left supramarginal gyrus (I^2^ = 1.79%; Harbord Egger bias tests p-value = 0.94) and right inferior parietal gyri (I^2^ < 0.01%; Harbord Egger bias tests p-value = 0.99) peaks showed low heterogeneity and nonsignificant small study effects.

**Table 2:** Effects of mind–body exercise on the functional connectivity of resting-state networks.

Brain regions with peak activation						Cluster breakdown	Resting-state network
Anatomical region	L/R	Total number of voxels	MNI coordinates	SDM-Z	p	Anatomical regions (Brodmann area)	
Mind–body exercise > physical activity							
Anterior cingulate/paracingulate gyri	L	11	−4, 42, −6	1.70	0.045	Anterior cingulate/paracingulate gyri (BA10)	DMN
Mind–body exercise < physical activity							
Supramarginal gyrus	L	109	−58, −42, 26	−1.85	0.033	Supramarginal gyrus (BA48) Superior temporal gyrus (BA42)	VAN

Analysis conducted with (1) seed location and (2) control group condition as covariates.

Significance threshold: uncorrected p < 0.05.

Regarding spontaneous neuronal fluctuations that are indicated by the fractional amplitude of low-frequency fluctuations (fALFF), five studies reported significant changes in fALFF after mind–body exercise. Three studies reported changes in fALFF in multiple regions in the frontal, temporal and hippocampal regions after several weeks of mind–body exercise^30,37,38,99^, and one study reported network-level changes in fALFF after more than 14 years of Tai Chi practice^39^.

### Can mind–body exercise modulate brain activities associated with task performance?

Seven studies reported mind–body exercise-associated changes in brain activation (indicated by changes in BOLD signals) and functional connectivity in practitioners when they performed cognitive and affective tasks (Table 1). Regarding changes in BOLD signals, both short-term and long-term yoga practice induce changes in brain changes in the frontoparietal areas when participants perform various affective tasks. For instance, long-term yoga practice was found to induce a decrease in the activity of the right dlPFC during emotional picture viewing and an increase in activity of the left vlPFC during the emotional Stroop task^29^, while short-term practitioners were shown to have increased activation in the postcentral gyrus, left superior parietal lobule and right anterior supramarginal gyrus during emotional video viewing tasks^70^. Changes in cortical and subcortical activation patterns were also evident when long-term yoga practitioners engaged in the color Stroop task and tasks with low (i.e., 1-back) and high (i.e., 2-back) working memory loads^71^. Some evidence has revealed that short-term Tai Chi practice induces changes in frontal activations during task switching^8^. Regarding changes in task-based FC, long-term yoga practice strengthens FC within the frontoparietal network during the color Stroop task. During working memory tasks, FC is strengthened between the PFC and various brain regions, including the parietal brain areas, cerebellum and insula, while it is reduced within parietal brain areas, cerebellum and insula^71^. Some evidence revealed that long-term Tai Chi practice induced stronger frontostriatal FC during a decision-making task embedded with emotional components^72^.

### Can mind–body exercise induce structural changes in the brain?

Eight studies showed that mind–body exercise induced structural changes in the brain, with 7 studies indexed these structural changes in terms of gray matter volume. Multiple studies showed that short-term (i.e., 3 months) Qigong (Baduanjin) practice promotes an increase in gray matter volume the right hemisphere, namely the right precentral and middle occipital gyri^74^, as well as in the right hippocampus^38,98^, while for long-term Tai Chi and Yoga practitioners^32,73^, the gray matter volume is increased in multiple cortical and subcortical areas, including frontal, temporal and occipital lobes, as well as limbic, parahippocampal areas and cerebellum. In contrast, short-term yoga practice is found to induce grey matter reduction in frontoparietal^75^, as well as temporal regions^76^. One study showed that long-term Tai Chi practice promotes the overall integrity and efficiency of the white matter network, as evidenced by an increase in the normalized clustering coefficient and the characteristic path length^31^.

### Certainty assessment

A summary of findings and certainty assessment was reported in Table 3. Overall speaking, the certainty of the evidence reported above was low, mainly due to small number of studies, indirectness and inconsistencies.

**Table 3:** Summary of findings and certainty assessment.

Outcome	No. of studies	Effect (SDM-Z)	Design	Risk of Bias	Inconsistency	Indirectness	Imprecision	Publication bias	Certainty of the evidence
Resting-state brain activity (seed to whole-brain rsFC)	8	1.70 (anterior cingulate); −1.85 (left supramar-ginal gyrus)	Cross-sectional studies; RCTs	No serious risk of bias	No serious inconsistencies	Serious (healthy controls only)	No serious imprecision	No serious publication bias	⊕ ⊕ ⊝ ⊝ Low (due to small number of studies and indirectness)
Resting-state brain activity (seed-based rsFC, other synchronization parameters)	9	Not pooled	Cross-sectional studies; RCTs	No serious risk of bias	Some inconsistencies	No serious indirectness	No serious imprecision	–	⊕ ⊕ ⊝ ⊝ Low (due to small number of studies and inconsistencies)
Resting-state brain activity (fALFF)	5	Not pooled	Cross-sectional studies; RCTs	No serious risk of bias	Some inconsistencies	No serious indirectness	No serious imprecision	–	⊕ ⊕ ⊝ ⊝ Low (due to small number of studies and inconsistencies)
Task-based brain activity	7	Not pooled	Cross-sectional studies; RCTs	Some risk of bias	Some inconsistencies	No serious indirectness	No serious imprecision	–	⊕ ⊕ ⊝ ⊝ Low (due to small number of studies and inconsistencies)
Structural change (grey and white matter volume)	8	Not pooled	Cross-sectional studies; RCTs	No serious risk of bias	Some inconsistencies	No serious indirectness	No serious imprecision	–	⊕ ⊕ ⊝ ⊝ Low (due to small number of studies and inconsistencies)

*rsFC* resting-state functional connectivity, *fALFF* fractional amplitude of low-frequency fluctuations.

## Discussion

By systematically synthesizing the current fMRI literature, this review aimed to examine the effects of mind–body exercise on brain activation, functional neural connections and structural changes in the brain. After performing a systematic literature search, 34 empirical studies were included for either narrative or meta-analytic review. The following results were obtained from the qualitative and quantitative analyses of fMRI studies: (1) mind–body exercise modulates the rsFC of the DMN, VAN and DAN; (2) mind–body exercise-induced changes in activation patterns in the frontal regions, as well as the changes in frontoparietal functional connections; (3) changes in spontaneous neuronal fluctuations at rest in frontal, temporal and hippocampal regions are evident in short-term mind–body practice practitioners; and 4) short-term Qigong and long-term Tai Chi/Yoga practice induces changes in the gray matter volume in various cortical and subcortical brain regions. The clinical implications of these results are discussed in the subsequent paragraphs.

Mind–body exercise modulates functional connections in the brain at rest, as evidenced by changes in correlations of BOLD signals between brain regions, regional homogeneity and nodal efficiency. Specifically, the functional connections in brain regions within the DMN were found to be enhanced when compared to control conditions, and importantly, the increase in activation of a brain region within DMN (i.e. right inferior parietal gyri) is shown to be significantly associated with years of mind–body practice. The DMN has long been suggested to be responsible for self-awareness^77^ and the coordination of task-positive networks^78^. Previous studies have shown that the functional connections between the DMN and task-positive functional networks increase only when an individual is preparing to perform cognitive tasks^79^. The capability of mind–body exercise to enhance rsFC within the DMN may imply that mind–body exercise might promote the readiness to perform goal-directed tasks of an individual. It is interesting to note that the SDM-Z value of the meta-regression is negative at the Y-axis, and one might be tempted to associate this with the possible attenuation effect on the well-documented age-related decline in the right inferior parietal gyri^80–82^. We recommend cautious interpretation of results given the number of studies are limited for the meta-regression. Further studies are needed to explore how mind–body exercises maintain the function of the parietal network and to examine the associations between behavioral improvements and mind–body exercise-induced changes in DMN.

The systematic review and meta-analysis revealed that mind–body exercise modulates the functional connections of dorsal (DAN) and ventral (VAN) attention networks at rest. The DAN and VAN are the two brain functional networks that are established to be responsible for attention and cognitive control^83,84^. While the DAN, a network that exerts top-town control, is responsible for regulating the processing of external stimuli in facilitation of the successful engagement of goal-directed behavior, the VAN is a “bottom-up” network that is responsible for the detection of behaviorally relevant stimuli^85^. These two networks interact with each other during attention shifting; specifically, the VAN interrupts the activation of DAN to allow an individual to reorient his attention to a new external stimulus^84^. As revealed by our results, the upregulation of DAN and downregulation of VAN at rest may imply that mind–body exercise reduces the reactivity towards external stimuli while also enhancing the ability to focus on goal-directed behaviors. Indeed, this postulation is consistent with previous findings showing that mindfulness-based interventions reduce reactivity to temptations (e.g. food cues)^86^ and enhance attentional control^87^ in practitioners.

Apart from resting-state neural connections, mind–body exercise also modulates brain activation patterns and functional connectivity when individuals perform cognitive/affective tasks. Notably, mind–body exercise specifically modulates task-related brain activation patterns in the frontal cortex and the functional connections between frontal and other brain regions. The effect of abnormal frontal lobe activation patterns on behaviors has been extensively studied in individuals with multiple neurodevelopmental, neuropsychiatric and neurodegenerative diagnoses, such as autism^88^, depression^89^, and dementia^90^. As revealed in the currently available literature, mind–body exercise appears to alter brain activation patterns specifically in the prefrontal brain regions, which signifies its applicability as a treatment for patients with frontal abnormalities. Indeed, previous clinical studies have provided positive empirical evidence supporting its applications^2,91^. Notably, the studies that investigated the effects of mind–body exercise on brain activity during active task performance are heterogeneous in terms of study designs. More studies involving similar cognitive/affective tasks are needed to support meta-analytic synthesis, which will help us further understand how mind–body exercise modulates task-based brain activities and functional connections.

In addition, spontaneous neuronal fluctuation at rest, as indexed by fALFF changes, is modulated by short-term mind–body exercise. Because fALFF has been shown to be associated with cognitive control abilities^92,93^, changes in fALFF in multiple brain regions may imply a network-based modulation of neuronal activities, although further investigations are needed to dissect the relationship between regional-specific fALFF changes and behavioral enhancement. Moreover, some evidence shows that short-term Qigong and long-term mind–body exercise may increase gray matter volume in various brain regions, including the frontal, temporal and occipital lobes, as well as limbic and parahippocampal areas and cerebellum. These brain regions are involved in various cognitive processes, which have been shown to shrink with increasing age^94,95^, and the reduction in volume is associated with age-related functional decline^96^. Long-term mind–body practice reverses age-related neural degeneration, implying that these exercises may play a role in delaying aging in the general population. The finding that short-term Qigong promotes grey matter volume changes in both frontal and hippocampal regions is encouraging; future studies should investigate the effects of long-term Qigong practice on grey matter increment. Future cross-sectional studies might consider studying the associations between years of training and changes in gray matter volume to determine the length of time participants must engage in mind–body exercise practice to yield observable changes in brain structure, and longitudinal studies may help address whether the observed changes in gray matter volume are long lasting.

### Limitations

To the best of our knowledge, this was the first study that comprehensively investigated the effects of mind–body exercise on brain activation, neural connectivity and brain structures by means of narrative synthesis and coordinate-based meta-analysis, yet several limitations were noted. Although we attempted to retrieve all possible published fMRI studies that investigated the effects of mind–body exercise on brain activities and narratively synthesized studies that did not report whole-brain rsFC between-group difference, only 13 experiments were included for coordinate-based meta-analysis, which limited the power of our analysis, resulting in small effect sizes observed in the meta-analysis. Regarding the study characteristics, although we attempted to control for the between-study heterogeneity by conducting covariate analyses in the meta-analysis, we were aware of the unaddressed heterogeneity across studies in both narrative synthesis and meta-analysis, for example the heterogeneity induced by the inclusion of both RCTs and cross-sectional studies, participants of different age and biological sexes. We encourage future neuroimaging studies examining the effects of mind–body exercise at the whole-brain level, such that larger-scale meta-analytic reviews that controls for the between-study heterogeneity could be performed, which would deepen our understanding about how mind–body exercise can promote functional changes in different regions of the brain. In addition, researchers may also consider investigating the specific effects of mind–body exercise on DMN, VAN, DAN and their behavioral correlates. Last but not least, it is critical to study the differential effects of meditation-only, conventional-exercise-only, and mind–body exercise on the brain in future studies.

## Conclusion

This review examined the neurobiological effects of mind–body exercise on brain activation, functional neural connections and structural changes in the brain. A systematic literature search yielded 34 relevant empirical studies that were included in the review, and data from 13 fMRI experiments were included in the meta-analysis. The results show that mind–body exercise modulates the rsFC of task-negative and attentional control networks, while also changes frontal activation patterns and frontoparietal functional connections during various cognitive tasks. Additionally, preliminary data show that short-term mind–body practice alters spontaneous neuronal fluctuations at rest in frontal, temporal and hippocampal regions, and short-term Qigong practice is further shown to induce both cortical and subcortical grey matter increment. We recommend that future studies include both neuropsychological and neurophysiological/neuroimaging techniques to further understand the neural mechanisms underpinning mind–body exercise.

## Supplementary Information

Supplementary Table S1.

Supplementary Table S2.

## Data Availability

Data extracted from included studies would be available upon reasonable request made to the corresponding author.
