# Supplementary material for: The neurobiological effects of mind–body exercise: a systematic review and meta-analysis of neuroimaging studies
Source: Sci Rep. 2023 Jul 6;13:10948. doi: 10.1038/s41598-023-37309-4 (PMC10326064; doi:10.1038/s41598-023-37309-4)
Supplement: Supplementary file 2 — Supplementary Table S2. [file 41598_2023_37309_MOESM2_ESM.docx]

**Table S2**

**Electronic database search (most recent search date: 10 June 2023)**

| **Information source** | Scopus |
| --- | --- |
| **Search strategy** | ( TITLE-ABS-KEY ( "mindful*" OR "mind-body" OR "yoga" OR "tai chi" OR "qigong" ) AND TITLE-ABS-KEY ( "magnetic resonance imaging" OR "functional magnetic resonance imaging" OR "MRI" OR "fMRI" ) ) |
| **No. of records** | 1133 |

| **Information source** | Embase |
| --- | --- |
| **Search strategy** | (mindful*:ti,ab,kw OR 'mind-body':ti,ab,kw OR 'yoga':ti,ab,kw OR 'tai chi':ti,ab,kw OR 'qigong':ti,ab,kw) AND ('magnetic resonance imaging':ti,ab,kw OR 'functional magnetic resonance imaging':ti,ab,kw OR mri:ti,ab,kw OR fmri:ti,ab,kw) |
| **No. of records** | 807 |

| **Information source** | ScienceDirect |
| --- | --- |
| **Search strategy** | Title, abstract or author-specified keywords: ("mindful" OR "mind-body" OR "yoga" OR "tai chi" OR "qigong") AND ("magnetic resonance imaging" OR "functional magnetic resonance imaging" OR "fMRI" OR "MRI") |
| **No. of records** | 155 |

| **Information source** | PubMed |
| --- | --- |
| **Search strategy** | ("mindful*"[Title/Abstract] OR "mind-body"[Title/Abstract] OR "yoga"[Title/Abstract] OR "tai chi"[Title/Abstract] OR qigong[Title/Abstract]) AND ("magnetic resonance imaging"[Title/Abstract] OR "functional magnetic resonance imaging"[Title/Abstract] OR "MRI"[Title/Abstract] OR "fMRI"[Title/Abstract]) |
| **No. of records** | 503 |
